# The physician in the face of death in the emergency room

**DOI:** 10.11606/S1518-8787.2018052000296

**Published:** 2018-04-04

**Authors:** Janaína de Souza Aredes, Karla Cristina Giacomin, Josélia Oliveira Araújo Firmo

**Affiliations:** I Instituto René Rachou. Fundação Oswaldo Cruz. Programa de Pós-Graduação em Saúde Coletiva. Belo Horizonte, MG, Brasil; II Secretaria Municipal de Saúde de Belo Horizonte. Belo Horizonte, MG, Brasil; III Instituto René Rachou. Fundação Oswaldo Cruz. Núcleo de Estudos em Saúde Pública e Envelhecimento. Belo Horizonte, MG, Brasil

**Keywords:** Physicians, psychology, Attitude to Death, ethnology, Emergency Medicine, Anthropology, Medical, Qualitative Research, Médicos, psicologia, Atitude Frente à Morte, etnologia, Medicina de Emergência, Antropologia Médica, Pesquisa Qualitativa

## Abstract

**OBJECTIVE:**

To analyze how physicians, as part of a sociocultural group, handle the different types of death, in a metropolitan emergency service.

**METHODS:**

This is an ethnography carried out in one of the largest emergency services in Latin America. We have collected the data for nine months with participant observation and interviews with 43 physicians of different specialties – 25 men and 18 women, aged between 28 and 69 years.

**RESULTS:**

The analysis, guided by the model of Signs, Meanings, and Actions, shows a vast mosaic of situations and issues that permeate the medical care in an emergency unit. The results indicate that physicians may consider one death more difficult than another, depending on the criteria: age, identification or not with the patient, circumstances of the death, and medical questioning as to their responsibility in the death process.

**CONCLUSIONS:**

For physicians, no death is easy. Each death can be more or less difficult, depending on different criteria that permeate the medical care in an emergency unit, and it reveals different social, ethical, and moral issues.

## RESUMO

**OBJETIVO:**

Analisar a forma como médicos, enquanto parte de um grupo sociocultural, lidam com diferentes tipos de morte, em um serviço de pronto socorro metropolitano.

**MÉTODOS:**

Trata-se de uma etnografia realizada em um dos maiores serviços de pronto socorro da América Latina. A coleta dos dados deu-se ao longo de nove meses de observação participante e entrevistas com 43 médicos de diferentes especialidades – 25 homens e 18 mulheres, entre 28 e 69 anos.

**RESULTADOS:**

À análise, guiada pelo modelo dos Signos, Significados e Ações, nota-se um vasto mosaico de situações e questões que medeiam o cuidado médico em uma unidade de pronto socorro. Os resultados apontaram que os médicos podem considerar uma morte mais difícil se comparada a outras, a depender de critérios: o etário; a identificação ou não com o paciente; as circunstâncias da morte e o questionamento médico quanto a sua responsabilidade no processo de morte.

**CONCLUSÕES:**

Para os médicos, nenhuma morte é fácil. Cada morte pode ser mais ou menos difícil, a depender de diferentes critérios que medeiam o cuidado médico em uma unidade de pronto socorro e revelam questões de ordem social, ética e moral das mais diversas.

## INTRODUCTION

Death has been delayed by the advances in biotechnological resources, the introduction of specific spaces for intensive care^1^, and, more recently, the implementation of the Brazilian Mobile Emergency Care Service (SAMU) – which is essential for the survival of victims of accidents and violence^2^ ^,^ ^3^. In the emergency care, the space to fight for life, it is up to the physician to avoid or postpone death^1^ ^,^ ^3^. However, depending on the severity of the lesions, neither the agility and the efficiency of the urgent and emergency care nor the entire biomedical apparatus are enough to prevent this outcome^2^. Urgency is understood as an acute situation with no imminent risk of failure of vital functions, while emergency refers to the medical finding of health conditions that entail imminent risk of failure of vital functions and which require immediate medical treatment^4^.

International research studies explore the concept of “saving lives”^5^, the rational use of therapeutic interventions^6^, and the values of life and health that mediate medical decisions^7^. In Brazil, studies address the medical criteria for patient prioritization in the emergency care^8^, deaths in intensive care units (ICU)^1^, the impact of violence on emergency care^9^, and the relationships among the professional categories in SAMU^10^.

However, we identified no studies in the literature consulted that include the perception of the physician in relation to death in the context of urgency and emergency. In view of these gaps, the objective of this study was to analyze how physicians, as part of a sociocultural group, handle the different types of death, in a metropolitan emergency service.

## METHODS

This qualitative research is based on a hermeneutical anthropological approach^11^, which emphasizes the meaning that social groups attribute to certain phenomena, considering the sociocultural context. This is an analysis of human relations based on a process of meaning together with the individual and collective perspectives^12^, based on the assumptions of medical anthropology. This context introduces a comprehensive analysis directed to the dynamics of illness, considers the interference of culture in the health and illness process, and proposes critics to the biomedical model of health^13^. All elements that involve the healthdisease process result from meanings and interpretations linked to social, psychological, and biological processes and they contribute to the understanding of the different factors that influence the formation of “medical realities”.

### Study Area and Population

This study is part of an ethnographic study^11^ that began in 2012, in one of the largest public emergency care services for polytrauma and burn victims in Latin America. We chose an urgent and emergency service because the physicians there handle a very diverse public whose deaths come from varied circumstances.

Located in the center of the city of Belo Horizonte, Brazil, and a reference for the entire state of Minas Gerais, the research area has 440 beds, of which 130 are for intensive care, of which 24 are dedicated for the care of severely disabled persons (Field Notes, 2013). With approximately 600 physicians and 50 residents, the study population consisted of physicians on duty in specific sectors for patients at risk of death or permanent disabilities.

### Data Collection and Analysis

This study was based on empirical observations and guided interviews with a semi-structured guide about the medical care in face of the limits of life and death in the hospital context. We collected the data for nine months with participant observation and voluntary interviews – during day and night shifts – with 43 physicians from different specialties – 25 men and 18 women, aged between 28 and 69 years. The selection used the snowball sampling^14^ and the final sample was regulated by the saturation criterion^15^. There were no refusals.

Data analysis was inductive and guided by the “Signs, Meanings, and Actions” model^12^ in order to know how these professionals think and act in relation to the different types of death present in their work in the emergency room. We started from the pragmatic horizon of individuals in order to identify and understand the conceptual logics aggregated to their actions, as well as the different factors that influence them^12^.

To ensure the anonymity of the interviewees, they were identified, respectively, by order of interview, medical specialty, gender (F for female, M for male), and age.

### Ethical Aspects

This study is part of the larger project named “*VIDAS EM RISCO: uma abordagem antropológica sobre as representações da morte entre médicos que trabalham em setores de urgência*,” approved by the Ethics Committees of the Universidade Federal de Minas Gerais (CAAE 03751612.0.0000.5149) and Fundação Hospitalar de Minas Gerais (Record CEP/ADC/FHEMIG 022/2012 – SIPRO 29128/2012-7). It is in accordance with Resolution 466/2012 of the National Health Council.

## RESULTS AND DISCUSSION

In the public health area, a multitude of identical medical and physical conditions can generate different care demands. Emergency rooms and corridors are often places where tension and haste prevail in favor of the lives of a very diverse audience, including astonished professionals, family members, police officers, patients of all ages, among others (Field Notes, 2013).

**Figure 1:**
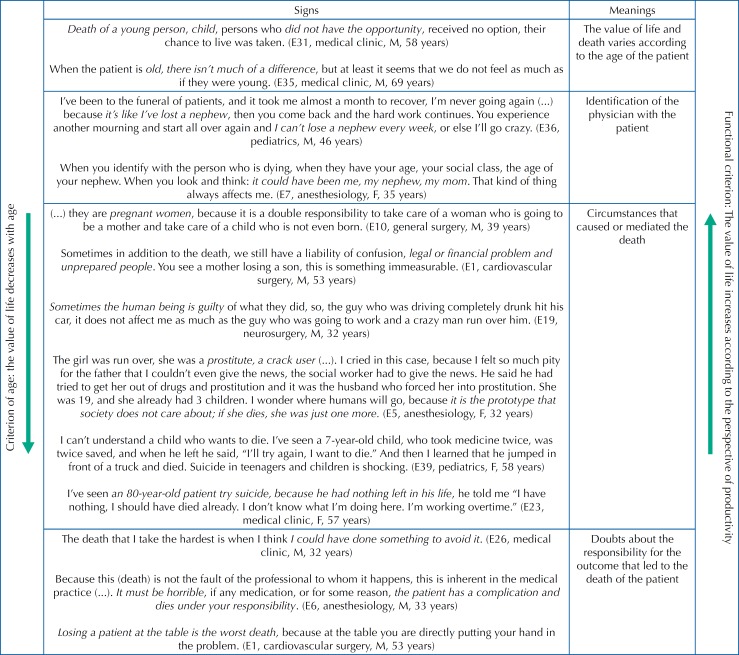
Criteria that guide the definition of the “most difficult” deaths for the physician.

The analysis of the ethnographic data allowed us to identify different emic criteria that guide the “most difficult” deaths for the physician in the researched context, namely: a) the criterion of age, b) identification or not with the patient, c) the circumstances of the death, and d) the medical questioning as to their responsibility in the death process, as summarized in Figure 1.

In the perception of this physician, the death of a child impacts the entire shift:

“We have no conditions, the whole room, that whole shift feels down when a child dies, it is very sad because it is not natural for a child to die (…)” (E22, medical clinic, M, 33 years)

At such times, there was a “chain commotion” throughout the hospital in the case of infant deaths – we often heard from the physicians: “I can't do it”, “I can't stand it”, “I have no structure for it” (Field Notes, 2013). Such reactions intensify the sense of defeat and failure that physicians experience in situations of infant death^1^. We can note that the difficulty of the physician is inversely proportional to the age of death of the patient, as in this statement:

“In the chronology of life, we are accustomed to bury the old and not the young.” (E38, pediatrics, F, 57 years)

**Figure 2:**
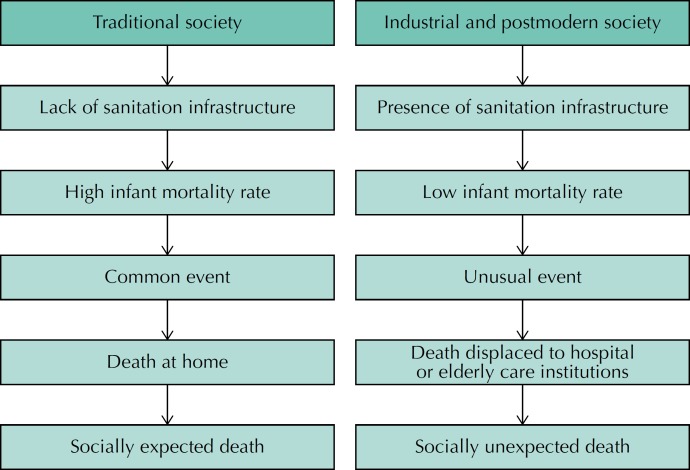
Evolution of the expectation of death in traditional and industrial and postmodern societies. Figure drawn by the authors, based on the studies of Ariès^16^.

In this perspective, the younger the patient, the higher is the chance of a favorable cure prognosis, the greater the potential for recovery, and the greater the investment in biomedical resources. On the other hand, death is more difficult. This perception differs from that observed by Ariès^16^ who has researched the place of the child and death in traditional and industrial societies, as shown in Figure 2.

In traditional society, child socialization, including the transmission of values and knowledge, was not guaranteed by the family, which weakened the bonds and reduced the social value of the life and death of the child^1^ ^,^ ^16^. As for infant death, it was a recurrent and premature event, from the lack of sanitation infrastructure, which happened at home, without any social repercussions. On the other hand, in the industrial and postmodern societies, the improvement of living conditions and the biotechnological progress of Medicine modified this situation and the death of children started to cause commotion and suffering^16^.

The criterion of age affects medical actions, that is, the orientation of the care also follows the logic of the course of life. Physicians of different specialties reflect on what affects the choice of one patient over another, should a vacancy arise in the ICU:

“If you have, for example, an 80-year-old patient with a very serious trauma, and I know that his chance to die is enormous, and I have a young man with the same trauma, and the chance to recover is a lot higher, if I have to decide, I'll opt for the young man. (…) Sometimes we are forced to make this type of decision.” (E10, general surgery, M, 39 years)

“When sending to the ICU, we seek the patient with greater possibility of recovery. There is this thinking that the CTI bed is very noble, very important, so the person who deserves this bed is the one that has a better condition to have a faster recovery, who has a perspective after discharge of returning to be a productive, fit person, and who can live his life normally. This is a pattern that is perhaps used all over the world.” (E21, medical clinic, M, 34 years)

Therefore, the expectation of physicians is translated into a full functional recovery (of all functions of the body) and social recovery (reestablishment of bonds – “family interaction”, “able to work, support the family, study”) of the individual. For the physician, the sign “noble bed” serves to justify the degree of investment (or not) in the care, based on chronological, biological, social, and functional parameters. Thus, age also becomes an argument that is both positive (younger persons have more chances) and negative (older persons are disregarded), in situations with scarce resources in public services^8^.

For Debert^17^, chronological age becomes an essential factor in the function of the modern Government of regulating the social body from the production of categories and hierarchization of populations. Several factors have led to the chronologization of life: the delimited separation of childhood, adolescence, adulthood, and old age can be analyzed as a response to economic changes – the transition from the domestic unit to the labor market. The course of modern life, especially the positive value attributed to youth, mirrors the Fordist logic, based on the economic productivity and the conditioning of the individual “to the rationalizing requirements of the social order.” On the other hand, the Government would be the master institution of the course of life, insofar as it regulates all its stages, from birth to death^17^.

Therefore, underlying the criterion of age, the investment in the care is privileged according to the greater perspective of the functionality of the patient. Functionality is related to all body functions, activities, and participation; while inability is related to disability, activity limitation, or restriction on social participation^18^.

The age and functional criteria cross all the other criteria. In the perception of the interviewees, the care is based on the perspective of the economic productivity and the functionality of the individual, especially as to their adequacy to the conditions of production. This reflects the precepts governing the Western capitalist organization, according to that society – and, by extension, the health system – should invest only in potentially productive persons^19^. In health, functionality is associated with positive characteristics while inability is usually linked to negative aspects, depending on the social and cultural context in which they occur^18^.

The new contemporary model of death^20^ – and life – reflects the change in the perception of these concepts, parallel to the economic change that has taken place in society. With the emergence of capitalism and, consequently, a new social order, the life of persons had to be remodeled to the new social structure. The expansion of the industrialization process and the demand for a greater supply of labor in the urban environment resulted in a strong rural exodus. Such changes caused unhealthy housing conditions and the unavailability of the family to care for the sick; therefore, the patients were taken to other places of care: the hospitals. The migration of the dying process to the hospital has led to a redefinition of death, especially the ethical and moral principles that permeate the practice of physicians. Death becomes medicalized, inscribed in institutional rules and routines that focus on medical competence and efficiency to maintain life^21^.

At the same time, death and those patients with no possibility of cure became hidden and rejected in a society that is increasingly endowed with scientific and technological progress^20^ and based on values of production and consumption^22^. In this society, those who fail to participate in the work, shopping, and leisure are abandoned, marginalized, and forgotten.

Another criterion is the identification or not with the dead patient (Figure 1). This physician recognizes it:

“We can't be hypocritical, I'm here in the emergency room and a patient comes in, the victim of a shooting, then you learn the story that he was a robber who was exchanging gunfire with the police. Of course as a physician the interventions are the same in any patient. Now, it's very different if I'm here on duty and we have a one-year-old kid who drowned in the pool at Grandma's house. Of course, they were two patients who arrived here at the hospital and died, but there are cases that affect you; so who is on the team and has a child at that age, they put themselves in that situation. You have a family that suffered a car accident, and in the accident the father and mother died and the child was left alone. Or a father who has lost a child, of course, we have an involvement, commotion, it is different. Nobody is heartless in a situation like this, but of course you have to exercise and work in a technical way, the situations you live can't interfere with the decisions you have to make, in your clinical reasoning, this can't happen.” (E14, general surgery, M, 47 years)

During the participant observation, we could note that the medical care and the technique for restoring life respect the priority dictated by the clinical and hemodynamic condition, regardless of who the patient is. However, there is a contradiction between the thinking and acting of the professional: the physician readily takes care of the patient without distinction of any type; however, at the same time, this professional makes value judgments that contradict the postulates of an impartial medical practice^23^. The bond, the relation, and the solidarity of the professional echo the social violence of the daily life of the service, depending on the profile of “good guy, victim, or bad gay” that the physician implies for each patient. In this sense, we can say that in the context of urgency and emergency, the technique and the physician-patient relation can be two actions of opposite meanings throughout the care^23^.

Regarding the circumstances of the death (Figure 1), some are considered to be more difficult than others, as these professionals point out:

“I always joke that I don't like to be on duty here during the holidays, because it is the time that families travel, take the road, and there will always be a family tragedy, mother, father, children involved in some kind of more serious accident.” (E11, general surgery, M, 39 years)

“Nature already defines that the older adult has to die first (…). Also, we get kids here who are totally healthy. (…) It is an aggression for us, it is the interruption of a life that still had a great potential.” (E3, anesthesiology, F, 42 years)

In the universe researched, except for older adults, the death of children, parents, young persons, and pregnant women are considered as “premature or unfair”. For physicians, the death of children or young persons is an abrupt interruption of the becoming, which refers to the full potential of realizing achievements in the future^1^. This physician emphasizes the consequences of these abrupt deaths:

“Abrupt death, violent death, traffic, murder, robbery (…), they leave very dramatic sequelae for those who stay.” (E31, medical clinic, M, 58 years)

As for violence-related deaths, this is a Brazilian public health problem^24^, because, in a vicious cycle, it is at the origin of the trauma – understood as any and every injury to the body, caused by an external action, of physical or chemical origin (Field Notes, 2013) – and at the obligation of the victim to handle possible sequelae that do not let him or her forget the cause of the suffering.

In the perception of the physician, death by suicide acquires distinct meanings depending on the stage of life of the patient. In childhood and youth, someone who wants to take their on life generates a sense of guilt at the impotence of the professional in addressing the suffering that he or she supposes that the person experiences. In the suicide of older adults, the lack of social bonds, hopelessness, and emotional distress^25^ that the professional attributes to old age make the act justifiable to some degree. We highlight the underreporting of the cases of suicide in this age group: in older adults, death is expected and many self-exterminations are attested as if they were accidents or deaths from natural causes^26^.

For the professionals interviewed, the older adult already experiences, in a way, a *death in life* (Field Notes, 2013), which is translated into a social death – that which occurs before the biological death and implies the loss of social roles with political and economic repercussions, as well as repercussions in health care and medicalization of life^27^. This condition is extended to all patients emically considered to have “chronic sequelae”: victims of trauma or clinical disease, resulting in chronic sequelae. These persons experience a continuous dependence on human help and on devices, in addition to social isolation and lack of possibility of interaction with the environment.

As shown in Figure 1, a death is considered difficult when the professional has doubts about his or her responsibility in the outcome. This professional explains it:

“Eventually, we come to the conclusion that something could have been done here (…). Not that we were late from sloppiness, malpractice, or negligence. In trauma, you have to think and act fast, and sometimes we can't define (the conduct) at that moment (…). Sometimes a conduct, taken two minutes later, makes a difference.” (E11, general surgery, M, 39 years)

Today, the progress of medicine allows the cure and treatment of numerous diseases, but deaths that professionals believe could be prevented or less painful are still present.

For every professional, regardless of the medical specialty and the time working in it, the worst death is the one that he or she has to handle. This shows the impact of the death of a patient on their lives^23^, in view of their reaction to the consciousness of finitude, from the very human condition. Now, death does not choose the moment, the type, or who it will take – children, young, old, poor and rich, whites and blacks, and in different ways, natural, violent, accidental, premature –, delegating to the physician a different reflection for each case^3^.

**Figure 3:**
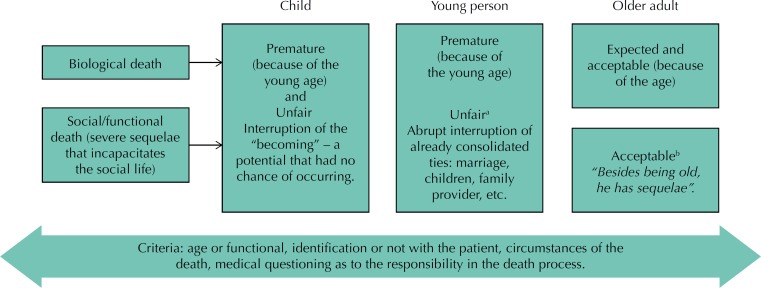
Death as an outcome of the care in the different stages of life. ^a^ Unfair: only in cases in which the patient is considered a “victim” of the trauma or illness. ^b^ Acceptable: because the survival of a severely disabled older adult is understood as a burden.

The analytical scheme of Figure 3 shows how physicians define death and how they justify it, according to the different aspects involved.

In the medical practice of emergency care, even if the universal access for citizens is sought, health systems, whether in developed or developing countries, have resource constraints, which should be considered in the formulation of rational decisions on therapeutic interventions^6^.

For Menezes^28^, the physician is responsible for decisions based on technical, social, and moral perspectives that have direct impacts on individuals (professionals and patients), institutions, and the community^8^. Another study^6^ states that the medical decision-making is summarily based on two types of judgment: the scientific and the social value. The first one is necessary to interpret science: how reliable is the effectiveness of a certain conduct? Are the results generalizable to the population in which the intervention could be used? While the second one is related to social sciences rather than to clinical sciences, and it proposes questions such as how can the life of children be considered more valuable than the life of their grandparents?^6^

There are no simple answers. Inserted in a specific professional field, which must on the one hand ensure life and on the other hand ethically react to the death of the patients, what we can perceive is a circular link between the condition of medical professional and subject^29^.

The thinking about the difficulties of the medical practice in view of death refers to the concept of total social fact proposed by Mauss^30^, which refers to the transversal understanding of different spheres of social life (psychological, physiological, cultural, historical, among others). We highlight that this totality in which social phenomena are inserted can only be apprehended in the life experience of the individual^30^.

Although this ethnographic analysis covered a myriad of types of death, we could not cover all the complexity that the phenomenon of death brings to physicians who work on time constraint, under strong personal, professional, and institutional pressure.

## FINAL CONSIDERATIONS

In emergency care, the involvement of the physicians interviewed varies according to the context of the death. The subjective position of the professionals – mediated by their perceptions and actions – is marked according to the moment of the course of life experienced by the patient. In this compartmentalized life cycle – child, young/adult, and older adult –, the medical care (and its outcome) is guided (and justified) in relation to the criterion of age and the potential functionality of the patient.

The result of the analysis shows that there are no easy deaths for physicians. They can be more or less difficult, depending on different criteria that permeate the medical care in an emergency unit, and they reveal different social, ethical, and moral issues.

Future research is needed to understand how physicians who work in other contexts and together with other types of death handle this phenomenon.

## References

[1] Menezes RA, Barbosa PC (2013). A construção da “boa morte” em diferentes etapas da vida: reflexões em torno do ideário paliativista para adultos e crianças. Cienc Saude Coletiva.

[2] Martins CBG, Mello Jorge MHP (2014). Analysis of service to fatal victims by external causes: domiciliary survey. Jl Nurs UFPE.

[3] Consorte J, Martins JS (1983). A morte na prática médica. A morte e os mortos na sociedade brasileira.

[4] Gomes AM (1994). Emergência: planejamento e organização da unidade: assistência de enfermagem.

[5] Chapell RY (2016). Against ‘Saving Lives’: equal concern and differential impact. Bioethics.

[6] Rawlins MD (2013). A population approach to the rational use of therapeutic interventions. Clin Ther.

[7] Watt H (2015). Life and health: a value in itself for human beings?. HEC Forum.

[8] Fortes PAC, Pereira PCA (2012). Priorização de pacientes em emergências médicas: uma análise ética. Rev Assoc Med Bras.

[9] Deslandes SF (2002). Frágeis deuses: profissionais da emergência entre os danos da violência e a recriação da vida.

[10] Seminotti EP, Neves EM (2014). Dos dramas de Narciso: reflexões antropológicas a partir de uma etnografia de um Serviço de Atendimento Móvel de Urgência (SAMU) de João Pessoa - PB. Ilha Rev Antropol.

[11] Geertz C (1989). A interpretação das culturas.

[12] Corin E, Uchôa E, Bibeau G, Koumare B (1992). Articulation et variations des systèmes de signes, de sens et d'actions. Psychopathol Afr.

[13] Good BJ, Del Vecchio Good MJ, Eisenberg L, Kleinman A (1980). The meaning of symptoms: a cultural hermeneutic model for clinical practice. The relevance of Social Science for Medicine.

[14] Patton MQ (2002). Qualitative research and evaluation methods.

[15] Fontanella BJB, Luchesi BM, Moretti B, Saidel MGB, Ricas J, Turato ER (2011). Amostragem em pesquisas qualitativas: proposta de procedimentos para constatar saturação teórica. Cad Saude Publica.

[16] Ariès P (1981). História social da criança e da família.

[17] Debert GG (2010). A dissolução da vida adulta e a juventude como valor. Horiz Antropol.

[18] (2004). Classificação Internacional de Funcionalidade, Incapacidade e Saúde - CIF.

[19] Bauman Z, Dentzien Plínio (2001). Modernidade líquida.

[20] Ariès P (1977). História da morte no ocidente: da Idade Média aos nossos dias.

[21] Herzlich C (1993). Os encargos da morte.

[22] Kamal TA, Martins JS (1983). A morte, o sobrenatural e a continuação da vida. A morte e os mortos na sociedade brasileira.

[23] Aredes JS, Modesto AL (2016). “Entre vidas e mortes, entre máscaras e fugas”: um estudo sobre a prática médica hospitalar. Physis.

[24] Murray J, Cerqueira DR, Kahn T (2013). Crime and violence in Brazil: systematic review of time trends, prevalence rates and risk factors. Aggress Violent Behav.

[25] Minayo MCDS, Cavalcante FG (2015). Tentativas de suicídio entre pessoas Idosas: revisão de literatura (2002/2013). Cienc Saude Coletiva.

[26] Deuter K, Procter N, Evans D, Jaworski K (2016). Suicide in older people: revisioning new approaches. Int J Mental Health Nurs.

[27] Gurgel WB (2007). A morte como questão social. Barbaroi.

[28] Menezes RA (2000). Difíceis decisões: uma abordagem antropológica da prática médica em CTI. Physis.

[29] Aredes JS (2012). De perto e de longe: um estudo sobre as representações da morte entre médicos de CTI. Rev Med Minas Gerais.

[30] Mauss M (1950). Sociologia e antropologia.

